# FTIR and GC–MS spectral datasets of wax from *Pinus roxburghii* Sarg. needles biomass

**DOI:** 10.1016/j.dib.2017.09.074

**Published:** 2017-10-11

**Authors:** Pallavi Dubey, Pradeep Sharma, Vineet Kumar

**Affiliations:** Chemistry Division, Forest Research Institute, Dehradun, India

## Abstract

The study has been carried out to investigate the chemical composition and type of linkages present in wax obtained from *Pinus roxburghii* Sarg. needles biomass. The spectroscopic techniques *viz*. FT-IR and GC–MS were employed to obtain spectral datasets. The results were analysed to identify major structural components constituting wax in native state. The spectral recordings were carried out at three different stages which include native wax, hydrolysed fatty acids and their corresponding methyl esters. Further, mass fragmentation has been discussed to represent the observed m/z values obtained in electron impact spectrum of fatty acid methyl esters.

**Specifications Table**

**Table:** 

Subject area	*Chemistry*
More specific subject area	*Spectroscopic studies for structural elucidation of plant wax*
Type of data	*Figure and table*
How data was acquired	***FTIR:** Perkin Elmer–Spectrum RX-I FTIR*
	***GC–MS:** 7890B Gas chromatograph coupled with 5977A mass spectrometer, equipped with electron impact (EI) and quadrupole analyser (Agilent Technologies, Santa Clara, CA, USA).*
Data format	*Analyzed*
Experimental factors	*a)* *Isolation, purification and saponification of wax* *b)* *Preparation of fatty acid methyl esters (FAME) for GC–MS analysis* *c)* *FTIR analysis of wax and hydrolysed fatty acids* *d)* *Separation, Identification and mass fragmentation analysis of fatty acid methyl esters*
Experimental features	*Wax was isolated from *Pinus roxburghii* needles.*
	*Isolated wax was saponified and its fatty acid methyl esters were prepared. The FTIR and GC–MS data was recorded and results were interpreted*.
Data source location	*Dehradun, India*
Data accessibility	*Data is included in this article*

**Value of the data**

• The spectral data values are significant to study structure of wax biopolymer isolated from *Pinus roxburghii* needles.
• The FTIR and GC–MS spectral values of samples *viz.* native wax, hydrolysed fatty acids and their corresponding fatty acid methyl esters were recorded successively in order to demonstrate systematic data sets of wax.
• The spectral datasets obtained from different spectroscopic techniques on correlation, lead to structural composition of native wax present in cuticular layer of needles. The study would provide a lead to researchers for future scientific investigations of plant wax.

## 1 Data

**Fig. 1.1:**
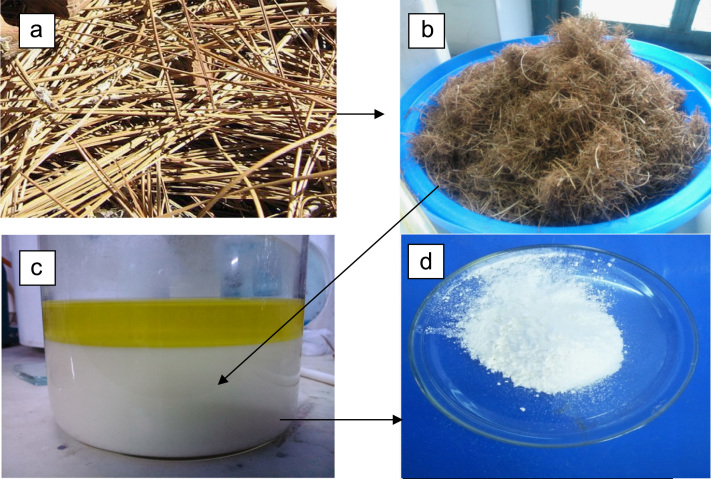
a) Pine needle biomass; b) Collected Plant material; c) Wax isolation d)Isolated and purified wax.
(ii) Fig. 1.2 illustrate the FT-IR spectrum of wax. In continuum, Fig. 1.3 indicate FT-IR spectrum of fatty acids obtained from saponification followed by acidification; Fig. 1.4 indicates FT-IR spectrum of fatty acid methyl esters.

The data which has been presented here include the following points:

(i) Fig. 1.1 explains different stages of the experiment starting from a) abundantly available Pine needle biomass; b) collected plant material; c) wax isolation; d) isolated and purified wax, thereby, demonstrating the stepwise process for isolation of wax for further analysis.

**Fig. 1.2:**
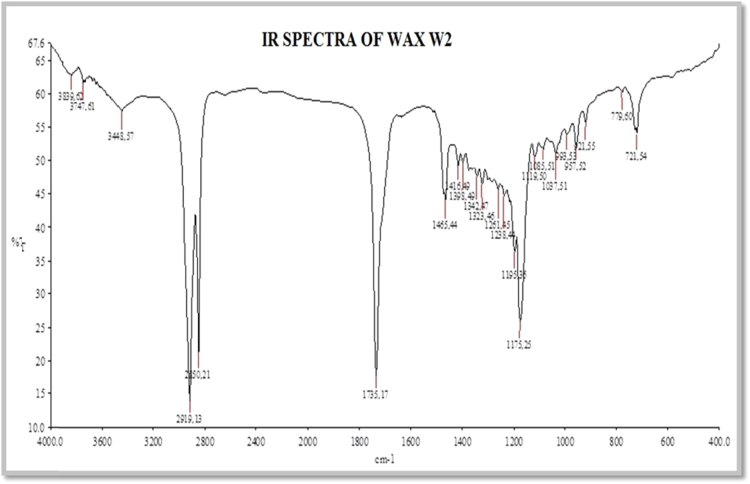
FT-IR spectrum of wax.

**Fig. 1.3:**
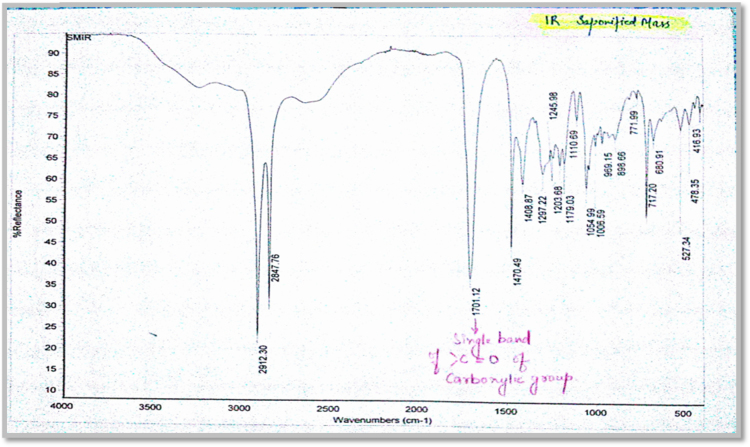
FT-IR spectrum of fatty obtained after saponification followed by acidification.

**Fig. 1.4:**
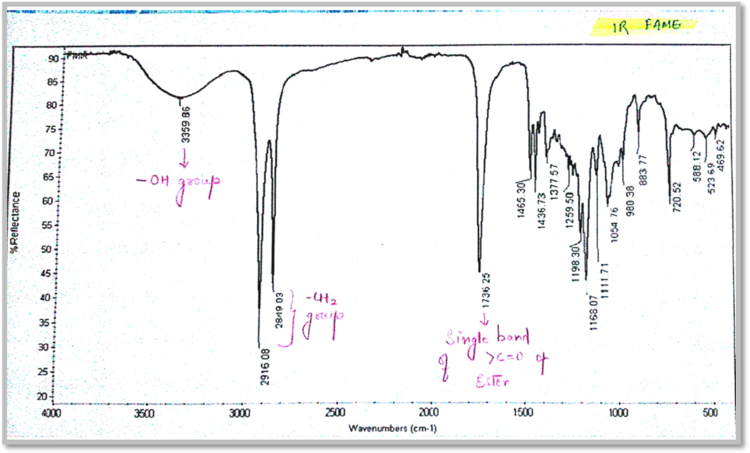
FT-IR spectrum of Fatty acid methyl esters.
(iii) Fig. 1.5 shows chromatogram obtained from GC–MS analysis of fatty acid methyl esters. Fig. 1.6, Fig. 1.7 represent mass spectrum of the peak obtained at RT 38.90 min and its mass fragmentation pattern respectively.

**Fig. 1.5:**
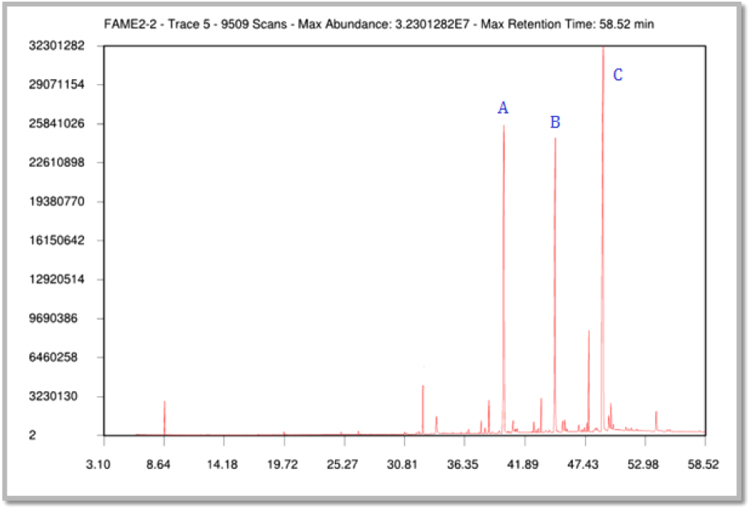
Chromatogram obtained from GC–MS analysis of Fatty Acid Methyl Esters.

**Fig. 1.6:**
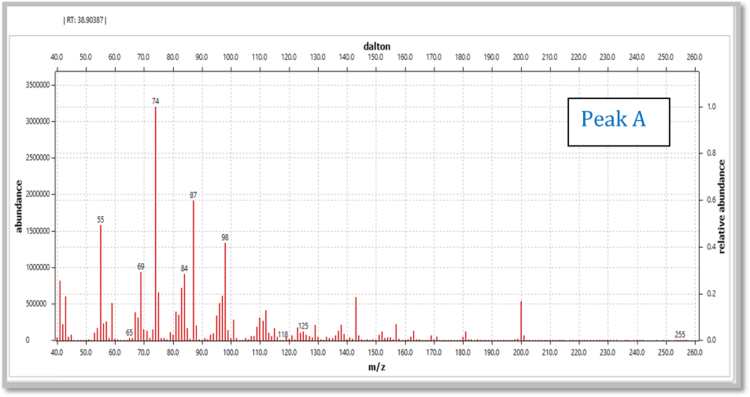
Mass spectrum of the peak obtained at RT value of 38.90 min.

**Fig. 1.7:**
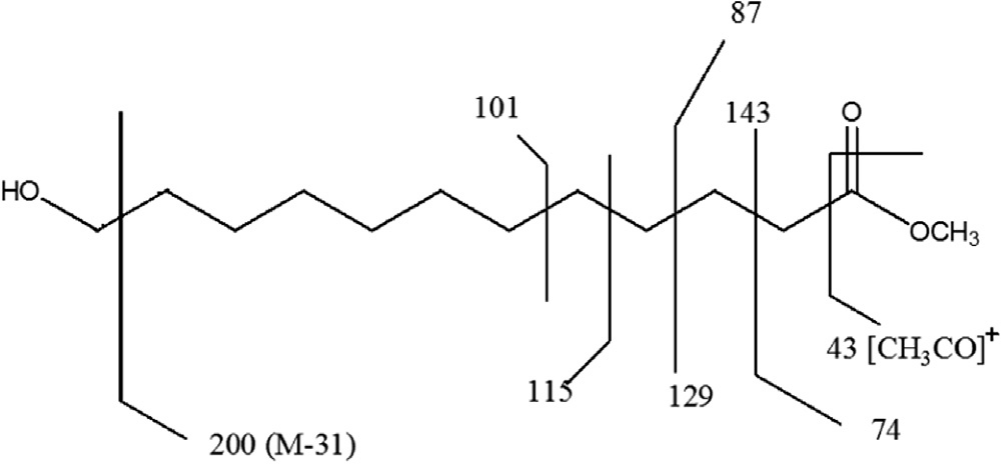
Mass fragmentation pattern of the compound representing chromatogram peak at RT value of 38.90 min.
(iv) Fig. 1.8, Fig. 1.9 show mass spectrum of the peak recorded at RT 43.86 min and corresponding mass fragmentation pattern.

**Fig. 1.8:**
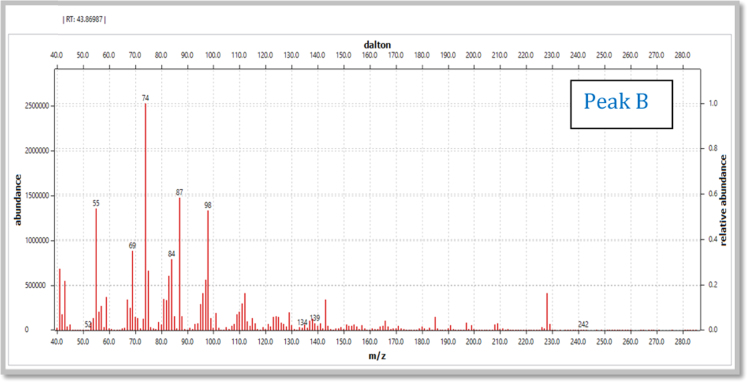
Mass spectrum of the peak recorded at the RT value of 43.86 min.

**Fig. 1.9:**
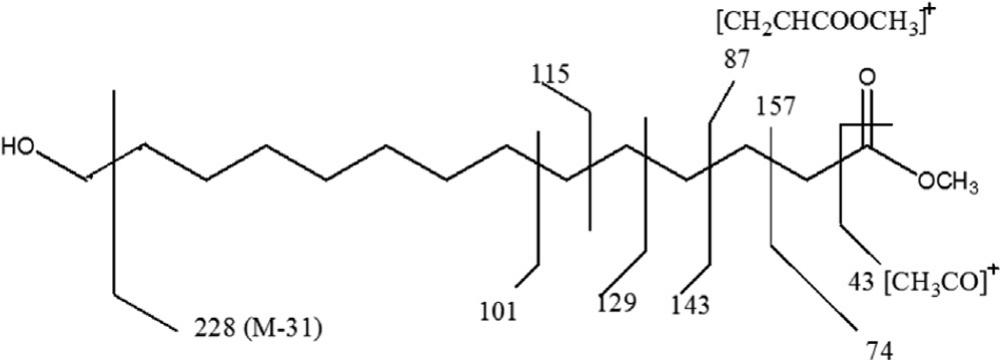
Mass fragmentation pattern of the compound representing chromatogram peak at RT value of 43.86 min.
(v) Fig. 1.10, Fig. 1.11 represent mass spectrum of peak observed at RT 48.46 minutes and its mass fragmentation pattern.

**Fig. 1.10:**
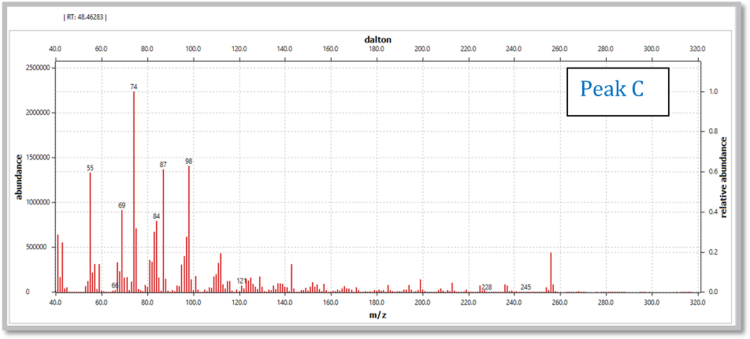
Mass spectrum of peak recorded at the RT value of 48.46 min.

**Fig. 1.11:**
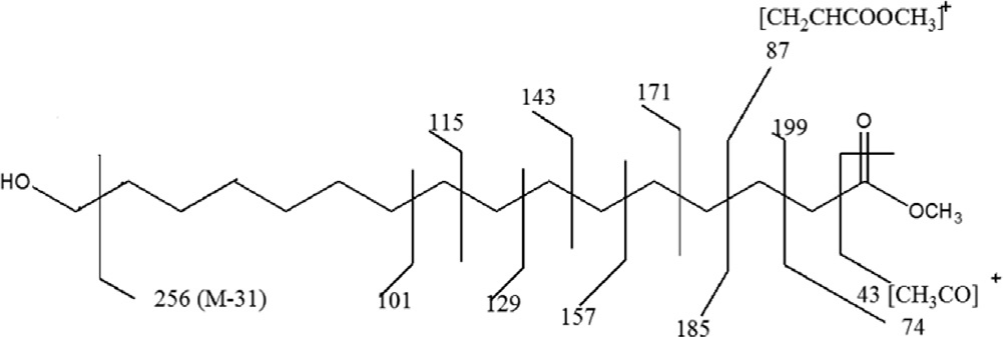
Mass fragmentation pattern of the compound representing chromatogram peak at RT value of 48.46 min.
(vi) Table 1.1 illustrate m/z values observed in mass spectrum indicating chromatogram peaks at the RT values 38.90, 43.86 and 48.46 minutes respectively.

**Table 1.1:** Mass fragmentation [1] and identified major peaks at the RT value of 38.90 min (A: Methyl-12-hydroxydodecanoate), 43.86 min (B: Methyl-14-hydroxytetradecanoate) and 48.46 min (C: Methyl-16-hydroxyhexadecanoate).

Fragmentation values of FAME peaks with respective retention time (RT) values in minutes																
RT 31.06		43	55	74	87	101	115	129	143	157	171	183	214			
RT 32.35			43	55	60	73	85	97	115	129	143	157	171	183	200	253
RT 37.54		45					74	98	112	129	143	163	180	197	213	229
RT 38.91	A	41	43	55	69	74	87	98	112	129	143	157	181	200		
RT 39.80		41	43	55	69	73	84	98	112	129	143	157	171	186	213	
RT 43.89	B	41	43	55	69	74	87	98	112	129	143	166	185	209	228	
RT 47.17		45	55	74	98	125	143	166	183	199	217	236	253	269	285	
RT 48.57	C	41	43	55	74	98	112	129	143	157	171	185	199	213	236	256

## 2 Experimental design, materials and methods

### 2.1 Collection of plant material

The plant material (fresh fallen mature pine needles) was collected during the month of April-May from *P. roxburghii* plantations at Forest Research Institute, Dehradun, India.

### 2.2 Fourier Transform Infrared spectroscopy

The samples were recorded using Perkin Elmer–Spectrum RX-I FTIR with a resolution of 1 cm^−1^ and scan range of 4000 cm^−1^ to 400 cm^−1^ using KBr pellet method.

### 2.3 Gas Chromatography - Mass Spectroscopy analysis

The GC–MS analyses were performed on a 7890B gas chromatograph coupled with 5977A mass spectrometer (Agilent Technologies, Santa Clara, CA, USA), equipped with electron impact (EI) ionization source and quadrupole mass analyser. The samples were injected in the split mode (split ratio of 80:1). The injector temperature was kept at 280 °C. The fused silica capillary column (DB-5ms, 30 m×250 µm; a film thickness of 0.25 µm) was used for separations. The carrier gas was helium, having a constant flow rate of 1.2 mL/min. The temperature program was maintained at initial temperature of 40 °C with a hold of 4 min, followed by increase of temperature of 4 °C /min up to final temp. of 220 °C with hold time of 5 min. The ion source temperature was 250 °C. Standard 70 eV EI spectra were recorded from 25 to 400 m/z mass range. The separated constituents in GC chromatogram were identified based on mass fragmentation and NIST library.
