# Supplementary material for: FTIR and GC–MS spectral datasets of wax from Pinus roxburghii Sarg. needles biomass
Source: Data Brief. 2017 Oct 11;15:615–22. doi: 10.1016/j.dib.2017.09.074 (PMC5655405; doi:10.1016/j.dib.2017.09.074)
Supplement: Supplementary file 1 — Supplementary material [file mmc1.pdf]

Conflicts of Interest Statement

Manuscript title: FTIR and GC-MS spectral datasets of wax from Pinus roxburghii Sarg. needle biomass

The authors whose names are listed immediately below certify that they have NO affiliations with or involvement in any organization or entity with any financial interest (such as honoraria; educational grants; participation in speakers' bureaus; membership, employment, consultancies, stock ownership, or other equity interest; and expert testimony or patent-licensing arrangements), or non-financial interest (such as personal or professional relationships, affiliations, knowledge or beliefs) in the subject matter or materials discussed in this manuscript.

Author names:

1. Pallavi Dubey
2. Dr. Pradeep Sharma
3. Dr. Vineet Kumar

The authors whose names are listed immediately below report the following details of affiliation or involvement in an organization or entity with a financial or non-financial interest in the subject matter or materials discussed in this manuscript. Please specify the nature of the conflict on a separate sheet of paper if the space below is inadequate.

Author names:

— None ————.

This statement is signed by all the authors to indicate agreement that the above information is true and correct:

Author's Name	Author's Signature	Date
1. Pallavi Dubey	Pallavi	October 16, 2017
2. Dr. Pradeep Sharma	Sharma	October 16, 2017
3. Dr. Vineet Kumar	Vineet Kumar	October 16, 2017
